# Seroprevalence of SARS-CoV-2 infection in pediatric patients in a tertiary care hospital setting

**DOI:** 10.1371/journal.pone.0310860

**Published:** 2024-09-24

**Authors:** Ploy Pattanakitsakul, Chanya Pongpatipat, Chavachol Setthaudom, Mongkol Kunakorn, Thiantip Sahakijpicharn, Anannit Visudtibhan, Nopporn Apiwattanakul, Surapat Assawawiroonhakarn, Uthen Pandee, Chonnamet Techasaensiri, Sophida Boonsathorn, Sujittra Chaisavaneeyakorn

**Affiliations:** 1 Faculty of Medicine Ramathibodi Hospital, Mahidol University, Bangkok, Thailand; 2 Chiangmai Ram Hospital, Chiangmai, Thailand; 3 Department of Pathology, Faculty of Medicine Ramathibodi Hospital, Mahidol University, Bangkok, Thailand; 4 Department of Pediatrics, Faculty of Medicine Ramathibodi Hospital, Mahidol University, Bangkok, Thailand; 5 Chakri Naruebodindra Medical Institute, Faculty of Medicine Ramathibodi Hospital, Mahidol University, Samut Prakan, Thailand; Chulalongkorn University Faculty of Medicine, THAILAND

## Abstract

Globally, cases of children’s coronavirus disease 2019 (COVID-19) have been reported since the pandemic started. Most children have an asymptomatic or mild infection. Therefore, the incidence rate of COVID-19 in children might have been underestimated. This study aimed to determine (1) the seroprevalence (and seroconversion rates) of COVID-19, including associated risk factors, in pediatric patients visiting hospitals; and (2) the immunological responses to COVID-19. This was a prospective, cross-sectional study. Patients aged 0–18 years who visited the hospital from September 2020 to February 2022 were included. Demographic, clinical, and laboratory data were reviewed. A total of 1,443 pediatric patients were enrolled. Of these, 323 (22.6%) had a history of COVID-19. In the pre-Delta period, the seroprevalence increased from 4.1% to 70.6% in all included patients and from 0.5% to 10% in patients without a known history of COVID-19 compared with the Delta-Omicron period. The seroconversion rate was 6.8% (19 per 100 person-years) in pediatric patients with COVID-19. Risk factors for COVID-19 seropositivity were respiratory symptoms, being in an outpatient department setting, and infection during the Delta-Omicron period. Exposure to household members with confirmed COVID-19 was a risk factor for seropositivity and seroconversion. Infection during the Delta-Omicron period and testing conducted >2 weeks after the onset of symptoms was associated with spike immunoglobulin (Ig) M and spike and nucleocapsid IgG, respectively. High nucleocapsid IgG levels were associated with pneumonia in pediatric patients with COVID-19. Pediatric patients exposed to household members with COVID-19 and respiratory symptoms should be tested for COVID-19. Nucleocapsid IgG can be used as a surrogate marker to identify patients who may have experienced pneumonia from COVID-19 and as a screening tool for the COVID-19 outbreak, regardless of COVID-19 vaccination status.

## Introduction

Coronavirus disease 2019 (COVID-19), caused by severe acute respiratory syndrome coronavirus 2 (SARS-CoV-2), can cause a broad spectrum of clinical illnesses, ranging from asymptomatic infection to critical illness with multiple organ failure and death [1]. As of December 2023, the number of globally confirmed cases of COVID-19 was >700 million, with approximately 7 million deaths [2]. There are still ongoing reports of new COVID-19 cases worldwide [2]. COVID-19 tests that can be performed at home are readily available, and there is a high rate of asymptomatic infection, which varies from 1.6 to 56.5% [3]. Therefore, the reported incidence rate of COVID-19 may be underestimated. The actual incidence rate of COVID-19 in children may not be ascertainable because most children with COVID-19 have an asymptomatic or mild infection. Furthermore, previous studies have reported that asymptomatic COVID-19 can cause transmission of COVID-19 [3, 4]. Therefore, seroprevalence estimates that can be used to assess the COVID-19 rate in patients showing minimal or no symptoms are useful for tracking and controlling the spread of the virus. In addition, studies on the SARS-CoV-2 seroprevalence and seroconversion among children are still limited.

Previous studies reported that successful mounting and persistence of the immune response to SARS-CoV-2 infection are crucial for controlling the infection and prevention of severe disease [5–7]. In children with COVID-19, a correlation between disease severity and anti-SAR-CoV-2 antibody response depends on the type of antibody responses against different SARS-CoV-2 antigens and SARS-CoV-2 strains as well as the kinetic of developing antibody responses [5, 7, 8]. After infection with COVID-19, infants and young children had longer durability of neutralizing antibodies against wild-type SARS-CoV-2 strain and SARS-CoV-2 spike-binding antibodies but similar durability of anti-nucleocapsid immunoglobulin (Ig) G response compared with adults. However, the kinetic reduction of antibody titers against SARS-CoV-2 variants of concern was similar in children and adults [5]. A study in a small group of pediatric participants showed a poor and delayed production of SARS-CoV-2 spike IgG during acute and convalescence phases in patients with severe COVID-19 [6]. Another study demonstrated lower anti-spike antibodies in pediatric participants hospitalized because of COVID-19 than those hospitalized for other conditions but incidentally found to have COVID-19 [9]. In addition, SARS-CoV-2 spike and nucleocapsid IgG levels were higher in pediatric participants with multisystem inflammatory response syndrome (MIS-C) than those without MIS-C. However, among pediatric participants with MIS-C, those who required intensive care units had lower SARS-CoV-2 spike and nucleocapsid IgG responses than those who did not [10]. The immune responses to SARS-CoV-2 infection are generally determined by the antibodies (immunoglobulin [Ig] M and G) developed against different SARS-CoV-2 antigens such as spike, nucleocapsid, and receptor-binding domain. However, the immune response to SARS-CoV-2 infection, especially in children, remains poorly understood.

This study aimed to assess the seroprevalence and seroconversion rates of COVID-19 in pediatric patients visiting the hospital to identify the associated risk factors for seropositivity, and to determine the SARS-CoV-2 antibody responses and their roles in predicting the prognosis.

## Materials and methods

Pediatric patients aged 0–18 years with or without a history of prior COVID-19 infection who visited an outpatient department (OPD) or acute respiratory infection clinic or were admitted to Ramathibodi Hospital, a tertiary-care university-based hospital in Bangkok, Thailand, were enrolled between September 1, 2020, and February 28, 2022. The outpatient visit included well-baby clinic, follow-up appointments with subspecialty clinics, or sick visits. For hospitalized patients, the reasons for admission included acute illness, elective surgery, or preparation for procedures. Pediatric patients with symptomatic human immunodeficiency virus infection, primary immune deficiency, chronic kidney disease, solid organ transplant, stem cell transplant within 2 years post-transplant, history of having rituximab within the past 6 months, receiving the induction phase of chemotherapy, or receiving blood component within the past 3 months were excluded from the study. Baseline demographic data including age, sex, body weight, height, body mass index z score, and comorbidities, were recorded. Epidemiological data, such as the risk of COVID-19 exposure, the reason for COVID-19 testing, and the history of COVID-19, were recorded. The clinical presentation, imaging results, laboratory data, and clinical outcomes were reviewed. Serum from all enrolled patients was tested for SARS-CoV-2 antibody using the Wondfo N total antibodies.

Patients without history of COVID-19 and had positive Wondfo N total antibodies were tested for nasopharyngeal SARS-CoV-2 using reverse transcriptase-polymerase chain reaction (RT-PCR) and a confirmed serological test including SARS-CoV-2 Spike (S) IgM, S IgG, and nucleocapsid (N) IgG. If there was no history of receiving a COVID-19 vaccine or receiving any first dose of COVID-19 vaccine <2 weeks before enrollment, patients who had at least one positive serology test (S IgM, S IgG, N IgG, or N total antibody test) were classified as having SARS-CoV-2 infection-induced seropositivity. If patient received the first dose of BNT162b2 >2 weeks before enrollment, those who had positive N IgG or N total antibodies were classified as having SARS-CoV-2 infection-induced seropositivity. An undetermined source of SARS-CoV-2 seropositivity included patients with at least one positive confirmed serology test in those who had received the inactivated COVID-19 vaccine >2 weeks before enrollment.

Patients with prior COVID-19 were further tested for S IgM, S IgG, and N IgG. Chest X-rays were obtained from all patients with confirmed COVID-19.

The study was approved by the Institutional Ethics Committee, Faculty of Medicine Ramathibodi Hospital, Mahidol University (COA.MURA2020/1033). The study protocol adhered to the tenets of the Declaration of Helsinki and Good Clinical Practice principles. Written informed consent was obtained from the parents or legal guardians of all participating children.

### Definitions

According to the guidelines issued by the Department of Disease Control in Thailand during March 2020 and December 2021, a patient under investigation was defined as having at least one of the following conditions [11, 12]: 1) a patient who had one of the following symptoms (a history of fever or body temperature ≥37.5°C, cough, runny nose, sore throat, tachypnea, dyspnea, difficulty breathing, anosmia, ageusia, red eyes, rashes, diarrhea) and, during the 14 days before the development of symptoms, had traveled to or from an area with a report of an ongoing COVID-19 outbreak, had worked or had close contact with tourists, had worked in a crowded place, had contact with many people, had contact with a confirmed patient or had contact with respiratory droplets of a suspected or confirmed case without appropriate protective equipment; 2) a patient who had pneumonia with a history of closed contact with a patient with COVID-19, had unexplained pneumonia that did not clinically improve within 48–72 hours, or had pneumonia with a profile consistent with COVID-19; and 3) a patient who was in a cluster of cases with similar respiratory tract symptoms within the same week and shared an epidemiological link.

The first, second, third, fourth, and fifth waves of the COVID-19 pandemic in Thailand were from March to November 2020, December 2020 to March 2021, April to June 2021, July to December 2021, and January to February 2022, respectively. The original strain (B.1.36.16) predominated in the first and second waves, whereas the Alpha (B.1.1.7), Delta (B.1.617.2), and Omicron (B.1.1.529) variants predominated in the third, fourth, and fifth waves, respectively [13]. The Omicron BA.1 variant (B.1.1.529) was predominated from January 2022 to February 2022, whereas the Omicron BA.2 variant (B.1.1.529) was predominated during early March 2022 [14]. The XBB, which is another subvariant of the Omicron, was first detected in February 2023 [15].

During the enrollment, the national school opening periods were from 1 July 2020 to 13 November 2020, 1 December 2020 to 4 April 2021, 14 June 2021 to 15 October 2021, and 1 November 2021 to 28 February 2022. Due to the second wave of the COVID-19 outbreak, some provinces, including Bangkok, were under a lockdown from 17 December 2020–7 February 2021.

The severity of COVID-19 was classified as mild, moderate, severe, or critical illness [1]. Mild illness was defined as upper respiratory tract symptoms not associated with dyspnea or abnormal radiological findings (non-pneumonia group). Moderate illness was defined as lower respiratory tract symptoms or abnormal radiological findings with oxygen saturation in room air >94%. The criteria for severe illness included oxygen saturation in room air <94%, tachypnea, and abnormal radiological findings involving >50% of the lung area. Critical illness was categorized as respiratory failure, shock, or multiorgan failure. The pneumonia group included moderate, severe, and critical cases.

Patients with a history of COVID-19 were defined as those with evidence of a positive nasal swab for SARS-CoV-2 by RT-PCR before enrollment by history or medical record. Patients with an immunocompromised state were classified as those who received chemotherapy, immunosuppressive agents, or underwent transplantation. Patients with comorbidities were defined as those with coexisting chronic medical conditions.

### Specimen collection and processing

Blood samples were collected and serology tests (Wondfo N total antibodies, Abbott S IgM, Abbott N IgG, and Ortho S IgG SARS-CoV-2 antibody tests) were performed. Nasopharyngeal swab specimens were collected and tested for SARS-CoV-2 using RT-PCR (SARS-CoV-2 Nucleic Acid Diagnostic Kit, Sansure Biotech Incorporation, Changsha, China) on the same day that blood samples were taken.

### SARS-CoV-2 serology assays

Serum from clotted blood specimens was tested for Wondfo SARS-CoV-2 N total antibodies and SARS-CoV-2 S IgM, S IgG, and N IgG. The Wondfo SARS-CoV-2 N total antibody assay (Guangzhou Wondfo Biotechnology Company Limited, Guangzhou, China), a lateral flow method, detects total antibodies against SARS-CoV-2 N protein. The Wondfo SARS-CoV-2 N total antibody test reported a positive result if there were two bands, one for control and one for total antibodies. The Abbott SARS-CoV-2 assay (Abbott Core Laboratory, Abbott Park, Illinois, USA), a chemiluminescent microparticle immunoassay, was used to detect S IgM and N IgG. SARS-CoV-2 S IgM and N IgG were reported as index values. They were interpreted as negative (index <0.6 for S IgM and <1.4 for N IgG) or positive results (index ≥0.6 for S IgM and ≥1.4 for N IgG) in accordance with the manufacturer’s cutoff index. The Vitros anti-SARS-CoV-2 assay (Ortho-Clinical Diagnostics, Bridgend, UK) is a chemiluminescent microparticle immunoassay that detects S IgG. SARS-CoV-2 S IgG was reported as an index value and was interpreted as a negative (index <1) or positive result (index ≥1) in accordance with the manufacturer’s cutoff index.

### SARS-CoV-2 RT-PCR assays

The extracted RNA from each patient’s nasopharyngeal and throat swab was used to perform SARS-CoV-2 testing by RT-PCR using two commercially available SARS-CoV-2 nucleic acid diagnostic kits. One kit (Sansure Biotech Incorporation, Changsha, China) targets open reading frames one a and b (*ORF1ab*) and nucleocapsid (N) gene fragments. The other kit (Seegene Incorporation, Seoul, South Korea) targets N, RNA-dependent RNA-polymerase and envelop gene fragments. These methods, including the internal control of each assay, were conducted in accordance with the manufacturer’s instructions.

### Statistical analysis

Baseline characteristics of the patients are reported as the mean (standard deviation, SD) for normally distributed data and as the median (interquartile range, IQR) for non-normally distributed data. Categorical data are reported as the frequency (percentage) and were compared using the chi-square test or Fisher’s exact test. The McNemar test was used to analyze paired categorical data. The Mann–Whitney U test was used to compare continuous variables. The potential factors associated with COVID-19 seropositivity were analyzed using a logistic regression. Cox proportional hazard regression was used to assess potential factors’ effect on the survival time simultaneously. Potential factors with *p*<0.10 in the univariable analysis were included in the multivariable analyses. The 95% confidence interval (95% CI) was also calculated. The Spearman rank correlation coefficient was used to assess the degree of correlation between antibody levels. Statistical significance was defined as *p*<0.05. The statistical analysis was performed using Stata/MP version 18 (Stata Corp., College Station, Texas, USA).

## Results

### Participants and sampling

**Fig 1:**
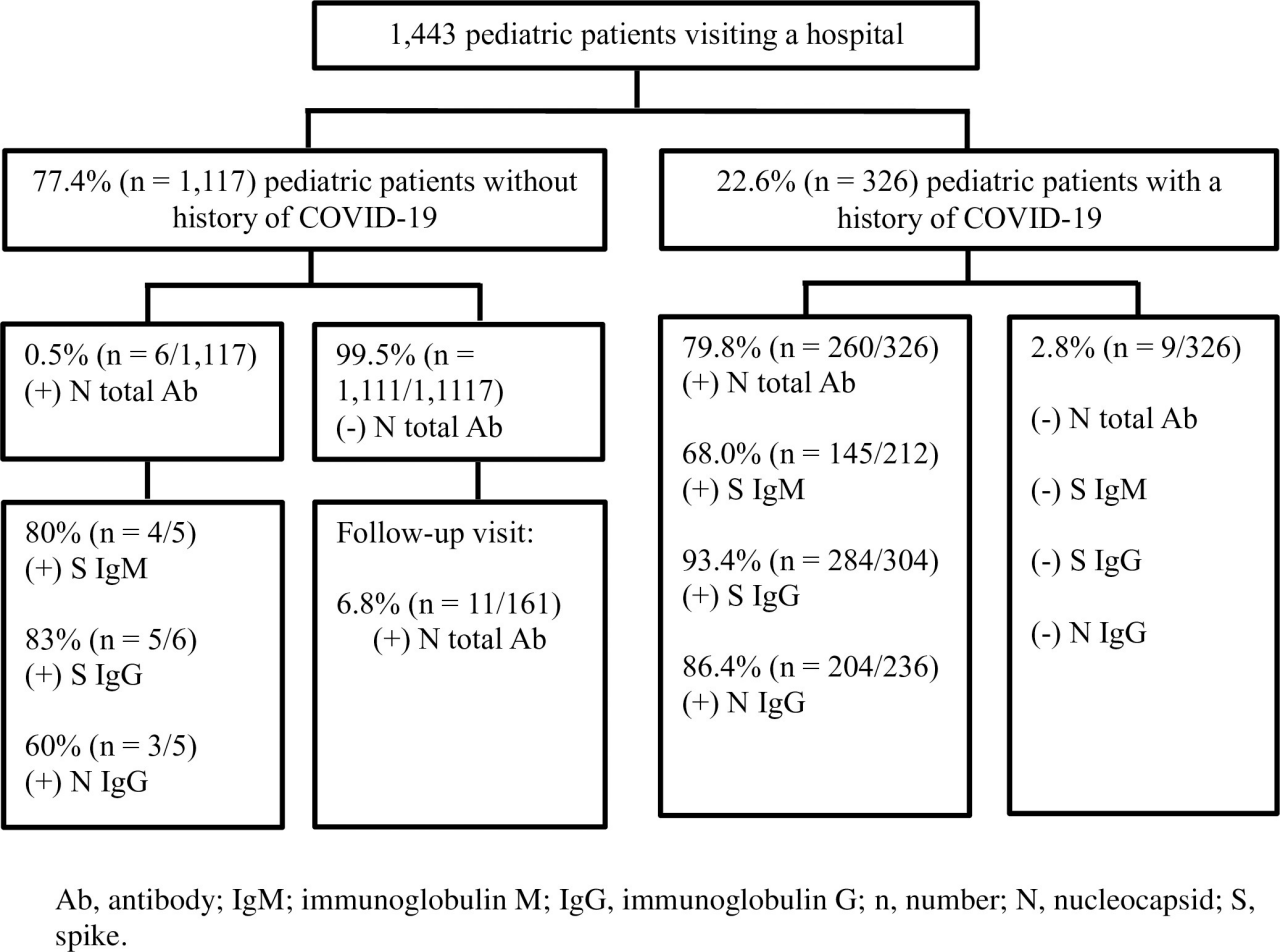
Flow chart summarizing serological tests of pediatric patients through study.

A total of 1,443 pediatric patients were enrolled from September 2020 to February 2022, including 1,117 (77.4%, 95% CI: 75.2%-79.5%) without and 326 (22.6%, 95% CI: 20.5%-24.8%) with COVID-19 before enrollment (Fig 1). The median age of the participants at enrollment was 5.0 years (IQR: 1.6–10.8) and 52.3% (755/1443, 95% CI: 49.7%-57.9%) were male participants (Table 1). All participants with prior COVID-19 had confirmation of COVID-19 by a positive nasal swab for SARS-CoV-2 by RT-PCR. Of the 1,117 patients without a history of COVID-19, 359 (32.1%, 95% CI: 29.4%-35.0%) had a negative nasal swab for SARS-CoV-2 by RT-PCR. The reasons for having a nasal swab to determine SARS-CoV-2 by RT-PCR were preoperative requirements (69.4%), having symptoms suspecting COVID-19 (29.8%), being a patient under investigation (0.5%), and concern by doctors (0.3%). Medical records and interview histories were used in the remaining patients to confirm COVID-19.

**Table 1:** Demographic data, clinical presentations, and potential factors associated with COVID-19 seropositivity.

Characteristic	Total (n = 1,443)	Seronegative (n = 1,177)	Seropositive (n = 266)	*P*	Univariable OR (95% CI)	*P*	Multivariable OR (95% CI)	*P*
Age, n (%)				<0.001				
< 1 year	257 (17.8)	234 (19.9)	23 (8.7)		Reference			
1–5 years	562 (39.0)	486 (41.3)	76 (28.6)		1.6 (1.0–2.6)	0.064	-	-
6–12 years	390 (27.0)	288 (24.5)	102 (38.4)		3.6 (2.2–5.8)	<0.001	-	-
13–18 years	234 (16.2)	169 (14.4)	65 (24.4)		3.9 (2.3–6.5)	<0.001	-	-
Male sex, n (%)	755 (52.3)	617 (52.4)	138 (51.9)	0.873	O.98 (0.75–1.28)	0.873	NA	NA
Comorbidities, n (%)	787 (54.5)	745 (63.3)	42 (15.8)	<0.001	0.1 (0.1–0.2)	<0.001	-	-
Immunocompromised, n (%)	173 (12.0)	172 (14.6)	1 (0.4)	<0.001	0.02 (0.003–0.158)	<0.001	-	-
Living in Bangkok*, n (%)	706 (48.9)	522 (44.4)	184 (69.2)	<0.001	2.8 (2.1–3.7)	<0.001	-	-
Family generation (≥3), n (%)	925 (64.3) n = 1,438	740 (63.1) n = 1,172	185 (69.6) n = 266	0.049	1.3 (1.0–1.8)	0.049	-	-
Household member (≥5), n (%)	742 (51.8) n = 1,433	589 (50.5) n = 1,167	153 (57.5) n = 266	0.038	1.3 (1.0–1.7)	0.038	-	-
Attending crowed area, n (%)	778 (53.9)	700 (59.5)	78 (29.3)	<0.001	0.3 (0.2–0.4)	<0.001	-	-
Public transportation, n (%)	332 (23.0)	303 (25.7)	29 (10.9)	<0.001	0.4 (0.2–0.5)	<0.001	-	-
Traveling abroad, n (%)	12 (0.8)	9 (0.8)	3 (1.1)	0.472	1.48 (0.40–5.51)	0.558	NA	NA
Exposure to household members with confirmed COVID-19, n (%)	318 (22.0)	67 (5.7)	251 (94.4)	<0.001	277.2 (155.8–493.3)	<0.001	42.4 (20.9–85.7)	<0.001
PUI	1,078 (74.7)	818 (69.5)	260 (97.7)	<0.001	19.0 (8.4–43.1)	<0.001	-	-
Received vaccine^†^, n (%)	8 (0.6)	0 (0.0)	8 (3.0)	<0.001	ND	ND	ND	ND
Outbreak wave^‡^, n (%)				<0.001		<0.001		0.001
Pre-Delta	1,135 (78.7)	1,088 (92.4)	47 (17.7)		Reference		Reference	
Delta-Omicron	308 (21.3)	89 (7.6)	219 (82.3)		55.6 (38.0–81.4)		3.0 (1.6–5.7)	
OPD setting, n (%)	455 (31.5)	209 (17.8)	246 (92.5)	<0.001	57.0 (35.3–92.0)	<0.001	5.0 (2.5–9.9)	<0.001
No symptom, n (%)	560 (38.8)	538 (45.7)	22 (8.3)	<0.001	0.1 (0.1–0.2)	<0.001	-	-
Fever, n (%)	542 (37.6)	403 (34.2)	139 (52.3)	<0.001	2.1 (1.6–2.8)	<0.001	-	-
Respiratory symptom, n (%)	566 (39.2)	355 (30.2)	211 (79.3)	<0.001	8.9 (6.4–12.3)	<0.001	2.1 (1.2–3.8)	0.008
GI symptom, n (%)	269 (18.6)	239 (20.3)	30 (11.2)	0.001	0.5 (0.3–0.7)	0.001	-	-

95% CI, 95% confidence interval; GI, gastrointestinal; IQR, interquartile range; NA, not applicable because of statistical insignificance at the univariable analysis level; ND, no data because of zero value of one comparator group; n, number; OPD, outpatient department; OR, odds ratio; PUI, patient under investigation; y, year;–(hyphen) factors not fit in the model during multivariable analysis.

* Capital city of Thailand where the incidence of COVID-19 was the highest.

^†^ COVID-19 vaccine (Pfizer or Sinopharm).

^‡^ Original strain (B.1.36.16) and alpha variant were the predominant circulating strains during the pre-delta (2^nd^ and 3^rd^) waves.

In this study, 1,832 serum samples were collected during the study period. The first hospital visit included 1,443 serum samples (78.8%, 95% CI: 76.8%-80.6%) from 1,443 pediatric patients. The number of serum samples collected from patients without COVID-19 who had more than one hospital visit during the study period was 389 (21.2%, 95% CI: 19.4%-23.2%) from 162 patients (14.6%, 95% CI: 12.6%-16.8%). The median number of hospital visits for patients without COVID-19 was two (IQR: 2–3).

### Seroprevalence of SARS-CoV-2 infection by screening with the N total antibody test

Overall, 18.4% (266/1443, 95% CI:16.5%-20.5%) of patients were positive for the SARS-CoV-2 N total antibodies by the Wondfo N total antibody test (Fig 1). The seroprevalence was 0.5% (6/1117, 95% CI: 0.20%-1.17%) and 79.8% (260/326, 95% CI: 75.0%-84.0%) in pediatric patients without and those with a history of COVID-19, respectively. Overall, the seroprevalence dramatically increased from 4.1% (47/1133, 95% CI: 3.1%-5.5%) during the pre-Delta period (first to third waves) to 70.6% (219/310, 95% CI: 65.2%-75.7%) during the Delta-Omicron period (fourth to fifth waves). The seroprevalence of children without (0.1%, 95% CI: 0.002%-0.52% vs. 10%, 95% CI: 4.24%-21.81%; *p*<0.001) and with a history of COVID-19 (69.7%, 95% CI: 57.1%-80.4% vs. 82.3%, 95% CI: 77.1%-86.7%; *p* = 0.023) significantly increased over the Delta-Omicron period compared with the pre-Delta period.

Of six patients without a history of COVID-19 and with a positive Wondfo N total antibody test, all of them had negative nasal swab for SARS-CoV-2 by RT-PCR, and five of the patients had at least two positive confirmed serological tests (SARS-CoV-2 S IgG, S IgM, or N IgG). Of the five patients classified as having SARS-CoV-2 infection-induced seropositivity, three had received the latest BNT162b2 with a median duration of 12.0 (IQR: 6.0–15.0) days before enrollment. One patient who did not have a complete confirmed serology test was defined as having SARS-CoV-2 infection-induced seropositivity because of no history of receiving the COVID-19 vaccine, having a history of a patient under investigation, and having N total antibody positivity. One patient was tested positive for all COVID-19 serological tests, which was unable to distinguish between infection-induced or vaccine-induced seropositivity owing to a history of receiving the inactivated BBIBP-CorV 29 days before enrollment (S1 Table).

### Potential factors associated with SARS-CoV-2 seropositivity by the N total antibody test

Sex and traveling abroad were not significantly different between the seropositive and seronegative groups (Table 1). Pediatric patients with SARS-CoV-2 seropositivity were significantly older than those with SARS-CoV-2 seronegativity (median: 8.5 years, IQR: 3.7–13.0 vs. median: 4.5 years, IQR: 1.4–10.1; *p*<0.001). The seropositive group had significantly higher percentages of living in Bangkok (*p*<0.001), ≥3 family generations (*p* = 0.049), ≥5 household members (*p* = 0.038), exposure to household members with confirmed COVID-19 (*p*<0.001), being a patient under investigation (*p*<0.001), receiving the COVID-19 vaccine (*p*<0.001), being in the Delta-Omicron wave (*p*<0.001), being in an outpatient department setting (*p*<0.001), fever (*p*<0.001), and respiratory symptoms (*p*<0.001) than the seronegative group. In contrast, the seropositive group had lower percentages of comorbidities (*p*<0.001), an immunocompromised state (*p*<0.001), attending crowded areas (*p*<0.001), using public transportation (*p*<0.001), no symptom (*p*<0.001), and gastrointestinal tract symptoms (*p* = 0.001) than the seronegative group. In multivariable analysis, patients who had exposure to household members with confirmed COVID-19 (odds ratio [OR]: 42.4; 95% CI: 20.9–85.7; *p*<0.001), those who were in an OPD setting (OR: 5.0; 95% CI: 2.5–9.9; *p*<0.001), those who had infection during the Delta-Omicron wave (OR: 3.0; 95% CI: 1.6–5.7; *p* = 0.001), and those who had respiratory symptoms (OR: 2.1; 95% CI: 1.2%-3.8%; *p* = 0.008) remained associated with COVID-19 seropositivity.

### Seroconversion rate of SARS-CoV-2 antibody and potential factors associated with seroconversion

**Fig 2:**
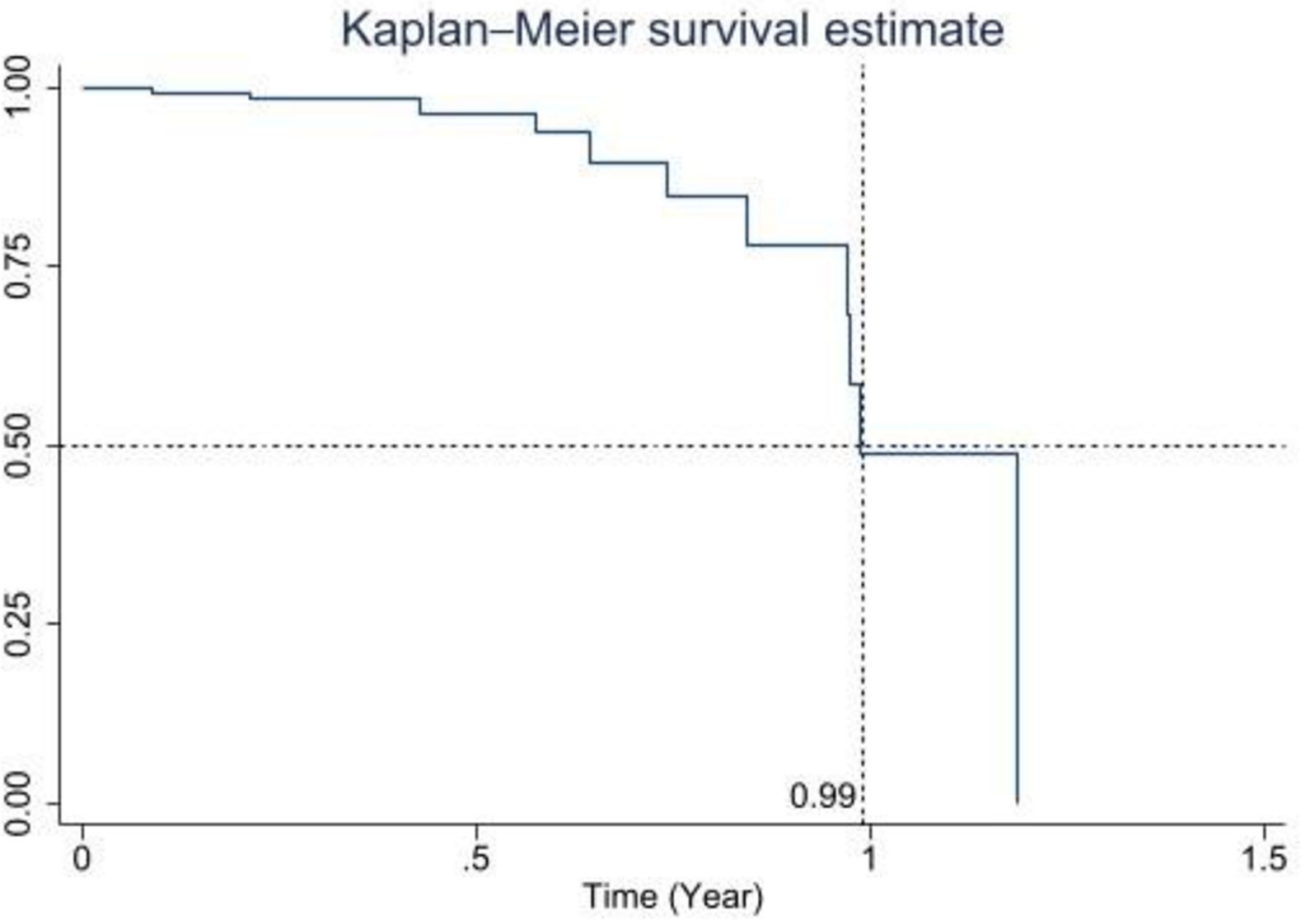
Kaplan-Meier curve of time to seroconversion in the samples of patients who had subsequent Wondfo total antibody tests. The vertical axis (y-axis) represents the seroconversion rate of the SARS-CoV-2 antibody. The horizontal axis (x-axis) represents time measured in years. The vertical dotted line indicates the median time to seroconversion of the SARS-CoV-2 antibody.

When the Wondfo N total antibody test was performed in 162 patients who were seronegative at enrollment and later tested from September 2020 to February 2022, 6.8% (11/162, 95% CI: 3.4%-11.8%) of them had seroconversion (Fig 1). The seroconversion rate was 19.0/100 person-years (95% CI: 10.5%-34.4%; 11 episodes in 57.8 person-years). The median time to seroconversion of the SARS-CoV-2 antibody was 0.99 years (Fig 2).

Potential associated factors for SARS-CoV-2 seroconversion were shown in Table 2. Sex, comorbidities, an immunocompromised state, living in Bangkok, type of family generation, number of household members, attending crowded areas, using public transportation, traveling abroad, COVID-19 outbreak period, fever, and gastrointestinal symptoms were not associated with SARS-CoV-2 seroconversion. After adjusting for having respiratory symptoms, being in an outpatient department setting, and outbreak period, SARS-CoV-2 seroconversion was associated with exposure to household members with confirmed COVID-19 (hazard ratio [HR]: 44.2; 95% CI: 4.88–399.3; *p* = 0.001) and age (1–5 years [HR: 0.01; 95% CI: 0.0002–0.15; *p* = 0.002], 6–12 years [HR: 0.02; 95% CI: 0.001–0.33; p = 0.00*7*], 13–18 years [HR: 0.06; 95% CI: 0.004–0.91; *p* = 0.043]).

**Table 2:** Potential factors associated with seroconversion of SARS-CoV-2 antibody.

Characteristic	Univariable HR (95% CI)	*P*	Multivariable HR (95% CI)	*P*
Age				
< 1 year	Reference		Reference	
1–5 years	0.07 (0.008–0.50)	0.009	0.01 (0.0002–0.15)	0.002
6–12 years	0.07 (0.007–0.64)	0.018	0.02 (0.001–0.33)	0.007
13–18 years	0.31 (0.043–2.24)	0.245	0.06 (0.004–0.91)	0.043
Male sex	0.45 (0.12–1.73)	0.244	NA	NA
Comorbidities	0.76 (0.16–3.64)	0.730	NA	NA
Immunocompromised status	0 (0)	1.000	NA	NA
Living in Bangkok*	2.01 (0.56–7.15)	0.282	NA	NA
Family generation (≥3)	0.48 (0.14–1.71)	0.261	NA	NA
Household member (≥5)	1.62 (0.44–6.01)	0.479	NA	NA
Attending crowed area	0.19 (0.02–1.64)	0.131	NA	NA
Public transportation	1.81 (0.33–9.76)	0.492	NA	NA
Traveling abroad	0 (0)	1.000	NA	NA
Exposure to household members with confirmed COVID-19	16.01 (3.09–82.98)	0.001	44.2 (4.88–399.3)	0.001
Outbreak wave^†^		0.095		
Pre-Delta	Reference			
Delta-Omicron	7.06 (0.71–70.06)		-	-
OPD setting	7.48 (1.81–30.9)	0.005	-	-
Fever	3.66 (0.72–18.66)	0.118	NA	NA
Respiratory symptoms	8.98 (2.30–35.09)	0.002	-	-
Gastrointestinal symptoms	0.45 (0.06–3.56)	0.447	NA	NA

HR, hazard ratios; GI, gastrointestinal; NA, not applicable because of statistical insignificance at the univariable analysis level; n, number; 95% CI, 95% confidence interval; OPD, outpatient department;–(hyphen) factors not fit in the model during multivariable analysis.

* Capital city of Thailand where the incidence of COVID-19 was the highest.

^†^ Original strain (B.1.36.16) and alpha variant were the predominant circulating strains during the pre-delta (2^nd^ and 3^rd^) waves.

### Seroprevalence, titer levels, and correlations of anti-SARS-CoV-2 antibodies in pediatric patients with COVID-19

Of 326 pediatric patients with COVID-19 confirmed by a positive nasal swab SARS-CoV-2 by RT-PCR, 79.8% (260/326, 95% CI: 75.0%-84.0%), 68% (145/212, 95% CI: 61.7%-74.6%), 93.4% (284/304, 95% CI: 90.0%-96.0%), and 86.4% (204/236, 95% CI: 81.4%-90.5%) were positive for N total antibodies, S IgM, S IgG, and N IgG, respectively (Fig 1). A total of 2.8% (9/326, 95% CI:1.3%-5.2%) of pediatric patients with COVID-19 were seronegative for all tested serological assays (Fig 1).

The ranges of COVID-19 seropositivity were 5–103, 5–191, 5–122, and 0–191 days after the onset of symptoms for S IgM, S IgG, N IgG, and N total antibodies, respectively. The median antibody levels were 1.9 index (IQR: 1.2–3.3), 9.2 index (IQR: 5.4–12.4), and 5.1 index (IQR: 3.6–6.4) for S IgM, S IgG, and N IgG, respectively. The number of seropositive patients detected by Wondfo N total antibodies was not significantly different from those detected by the chemiluminescent microparticle immunoassay for N IgG antibodies (McNemar test; *p* = 0.095). In addition, N IgG levels were strongly correlated with S IgG seropositivity (Spearman’s rho = 0.6947, *p*<0.001). However, S IgM levels were moderately correlated with N IgG levels (Spearman’s rho = 0.3335, *p*<0.001) and S IgG levels (Spearman’s rho = 0.3002, *p*<0.001) (S1 Fig).

### Pediatric patients with confirmed COVID-19 and their potential factors associated with positive anti-SARS-CoV-2 antibodies

A total of 202 (62.0%, 95% CI: 56.5%-67.3%) patients who had all serological tests (N total antibodies, S IgM, S IgG, and N IgG) were included in the analysis (Table 3). All patients had no history of receiving a vaccination against COVID-19 and were not an immunocompromised host. Age, sex, comorbidities, fever, gastrointestinal symptoms, and clinical severity were not significantly different between the S IgM, S IgG, N total antibody, and N IgG seronegative and seropositive groups (Table 3). The patients with S IgM seropositivity had a lower rate of infection during the Delta-Omicron period (*p* = 0.004) but a higher rate of having respiratory symptoms (*p* = 0.026) than those with S IgM seronegativity. The patients with S IgG seropositivity had a higher rate of infection during the Delta-Omicron wave (*p* = 0.016) and being in an OPD setting (*p*<0.001), but a lower rate of having no symptom (*p* = 0.015) than those with S IgG seronegativity. A higher percentage of patients with seropositivity of N IgG and N total antibodies were in an OPD setting than those with seronegativity of N IgG (*p* = 0.008) and N total antibodies (*p*<0.001). The median duration of serological testing after the onset of symptoms was longer in the S IgM seronegative group than in the S IgM seropositive group (46.0, IQR: 40.0–53.0 days vs. 38.5, IQR: 28.0–47.5 days; *p* = 0.0002) but shorter in S IgG seronegative group than in the S IgG seropositive group (13.0, IQR: 9.0–44.0 days vs. 43.0, IQR: 33.0–48.0 days; *p* = 0.0006). At <14 days after the onset of symptoms, patients with seropositivity of N total antibodies had the highest rate (6.5%) of positivity, whereas patients with S IgG seropositivity had the lowest rate (1.0%). At >14 days after the onset of symptoms, > 90% of patients had S IgM (97.1%), S IgG (99.0%), N IgG (98.9%), and N total antibodies (93.5%) seropositivity.

**Table 3:** SARS-CoV-2 antibodies in pediatric patients with COVID-19.

Characteristic	S- IgM (+) n = 140 (69.3)	S-IgM (-) n = 62 (30.7)	*P*	S-IgG (+) n = 191 (94.6)	S-IgG (-) n = 11 (5.4)	*P*	N-IgG (+) n = 178 (88.1)	N-IgG (-) n = 24 (11.9)	*P*	N Total (+) n = 168 (83.2)	N Total (-) n = 34 (16.8)	*P*
Age, n (%)			0.413			0.408			0.642			0.742
<1 year	12 (8.6)	5 (8.1)		15 (7.9)	2 (18.2)		14 (7.9)	3 (12.5)		13 (7.7)	4 (11.8)	
1–5 years	38 (27.1)	24 (38.7)		60 (31.4)	2 (18.2)		57 (32.0)	5 (20.8)		51 (30.4)	11 (32.4)	
6–12 years	55 (39.3)	19 (30.7)		71 (37.2)	3 (27.3)		65 (36.5)	9 (37.5)		64 (38.1)	10 (29.4)	
13–18 years	35 (25)	14 (22.6)		45 (23.6)	4 (36.4)		42 (23.6)	7 (29.2)		40 (23.8)	9 (26.5)	
Male sex, n (%)	73 (52.1)	32 (51.6)	0.945	98 (51.3)	7 (63.6)	0.426	92 (51.7)	13 (54.2)	0.819	92 (54.8)	13 (38.2)	0.079
Comorbidities, n (%)	26 (18.6)	9 (14.5)	0.482	33 (17.3)	2 (18.2)	0.939	32 (18.0)	3 (12.5)	0.506	27 (16.1)	8 (23.5)	0.295
Outbreak wave*, n (%)			0.004			0.016			0.928			0.741
Pre-delta	42 (30.0)	7 (11.3)		43 (22.5)	6 (54.5)		43 (24.2)	6 (25.0)		40 (23.8)	9 (26.5)	
Delta-omicron	98 (70.0)	55 (88.7)		148 (77.5)	5 (45.5)		135 (75.8)	18 (75.0)		128 (76.2)	25 (73.5)	
OPD setting, n (%)	126 (90.0)	56 (90.3)	0.944	177 (97.3)	5 (45.5)	<0.001	164 (92.1)	18 (75.0)	0.008	157 (93.45)	25 (73.5)	<0.001
No symptom, n (%)	11 (7.9)	5 (8.1)	0.960	13 (6.8)	3 (27.3)	0.015	13 (7.3)	3 (12.5)	0.376	155 (92.3)	31 (91.2)	0.831
Fever, n (%)	64 (45.7)	37 (59.7)	0.067	98 (51.3)	3 (27.3)	0.121	90 (50.6)	11 (45.8)	0.664	84 (50.0)	17 (50.0)	1.000
Respiratory symptoms, n (%)	120 (85.7)	45 (72.6)	0.026	34 (17.8)	3 (27.3)	0.430	146 (82.0)	19 (79.2)	0.734	140 (83.3)	25 (73.5)	0.178
GI symptoms, n (%)	17 (12.1)	3 (4.8)	0.109	20 (10.5)	0 (0.0)	0.258	18 (10.1)	2 (8.3)	0.784	18 (10.7)	2 (5.9)	0.390
Clinical severity, n (%)			0.949			0.156			0.076			0.858
Non-pneumonia	101 (72.1)	45 (72.6)		136 (71.2)	10 (90.9)		125 (70.2)	21 (87.5)		121 (72.0)	25 (73.5)	
Pneumonia	39 (27.9)	17 (27.4)		55 (28.8)	1 (9.1)		53 (29.8)	3 (12.5)		47 (28.0)	9 (26.5)	
Duration^†^, median (IQR) days	38.5 (28.0–47.5)	46 (40.0–53.0)	0.0002	43 (33.0–48.0)	13 (9.0–44.0)	0.0006	42 (31.0–48.0)	44 (14.5–50.5)	0.615	43 (31.0–48.0)	43.5 (28.0–52.0)	0.978
Titer, median (IQR)	2.0 (1.2–3.3)	0.3 (0.2–0.5)	<0.001	9.2 (5.4–11.7)	0.08 (0.01–0.34)	<0.001	5.1 (3.7–6.5)	0.4 (0.1–0.8)	<0.001	ND	ND	NA
Positivity, n (%)			0.227			<0.001			<0.001			0.002
< 14 days	4 (2.9)	4 (6.5)		2 (1.0)	6 (54.5)		2 (1.1)	6 (25.0)		11 (6.5)	8 23.5)	
≥ 14 days	136 (97.1)	58 (93.5)		189 (99.0)	5 (45.5)		176 (98.9)	18 (75.0)		157 (93.5)	26 (76.5)	

GI, gastrointestinal; IQR, interquartile range; n, number; NA, not applicable; ND, no data; N IgG, nucleocapsid immunoglobulin; N total, nucleocapsid total antibody; OPD, outpatient department; S IgM, spike immunoglobulin M; S IgG, spike immunoglobulin G.

* Original strain (B.1.36.16) and alpha variant were the predominant circulating strains during the pre-delta (2^nd^ and 3^rd^) waves.

^†^ Days after COVID-19 diagnosis or onset of symptom

S IgM seropositivity was negatively associated with the Delta-Omicron outbreak period (OR: 0.3; 95% CI: 0.1–0.7, *p =* 0.009) after adjusting for fever and respiratory symptoms (Table 4). After adjusting for the OPD setting, the Delta-Omicron outbreak period, and having no symptom, S IgG seropositivity was associated with testing conducted >2 weeks after the onset of symptoms (OR: 113.4; 95% CI: 18.2–707.1; *p*<0.001). In addition, testing conducted >2 weeks after the onset of symptoms was associated with N IgG seropositivity (OR: 29.3; 95% CI: 5.5–156.2, *p*<0.001) after adjusting for clinical severity and the OPD setting. N total antibody seropositivity was associated with the OPD setting (OR: 5.1; 95% CI: 1.9–13.6, *p* = 0.004) after adjusting for sex and the time of testing.

**Table 4:** Potential factors associated with S IgM, S IgG, N IgG, and N total antibody seropositivity.

	S IgM Uni OR (95% CI)	*P*	S IgM Multi OR (95% CI)	*P*	S IgG Uni OR (95% CI)	*P*	S IgG Multi OR (95% CI)	*P*	N IgG Uni OR (95% CI)	*P*	N IgG Multi OR (95% CI)	*P*	N Total Uni OR (95% CI)	*P*	N Total Multi OR (95% CI)	*P*
Sex, n (%)	1.0 (0.6–1.9)	0.945	NA	NA	0.6 (0.2–2.1)	0.430	NA	NA	0.9 (0.4–2.1)	0.819	NA	NA	2.0 (0.9–4.1)	0.082	-	-
OPD setting, n (%)	1.0 (0.4–2.6)	0.944	NA	NA	15.2 (4.1–56.0)	<0.001	-	-	3.9 (1.3–11.4)	0.013	-	-	5.1 (1.9–13.6)	0.001	5.1 (1.9–13.6)	0.***001***
Outbreak wave*, n (%)																
Pre-delta	Ref		Ref		Ref				Ref				Ref			
Delta-omicron	0.3 (0.1–0.7)	0.006	0.3 (0.1–0.7)	0.009	4.1 (1.2–14.2)	0.024	-	-	1.0 (0.4–2.8)	0.928	NA	NA	1.2 (0.5–2.7)	0.741	NA	NA
Asymptomatic infection, n (%)	1.0 (0.3–3.1)	0.960	NA	NA	5.1 (1.2–21.7)	0.026	-	-	1.8 (0.5–6.9)	0.382	NA	NA	1.2 (0.3–4.3)	0.831	NA	NA
Fever, n (%)	0.6 (0.3–1.0)	0.068	-	-	2.8 (0.7–10.9)	0.136	NA	NA	1.2 (0.5–2.8)	0.664	NA	NA	1.0 (0.5–2.1)	1.000	NA	NA
Respiratory symptoms, n (%)	2.3 (1.1–4.7)	0.028	-	-	1.7 (0.4–6.9)	0.435	NA	NA	1.2 (0.4–3.5)	0.734	NA	NA	1.8 (0.8–4.3)	0.182	NA	NA
Clinical severity, n (%)																
Non-pneumonia	Ref				Ref				Ref				Ref			
Pneumonia	1.0 (0.5–2.0)	0.949	NA	NA	4.0 (0.5–32.3)	0.188	NA	NA	3.0 (0.8–10.4)	0.088	-	-	1.1 (0.5–2.5)	0.858	NA	NA
Duration^†^, n (%)																
< 14 days	Ref				Ref		Ref		Ref		Ref		Ref			
≥ 14 days	2.3 (0.6–9.7)	0.239	NA	NA	113.4 (18.2–707.1)	<0.001	113.4 (18.2–707.1)	<0.001	29.3 (5.5–156.2)	<0.001	29.3 (5.5–156.2)	<0.001	4.4 (1.6–11.9)	0.004	-	-

GI, gastrointestinal; IQR, interquartile range; NA,not applicable because of statistical insignificance at the univariable analysis level; n, number; N IgG, nucleocapsid immunoglobulin G; Multi, multivariable; N total, nucleocapsid total antibody; Ref, reference; S IgM, spike immunoglobulin M; S IgG, spike immunoglobulin G; Uni, univariable;—(hyphen) factors not fit in the model during multivariable analysis

* Original strain (B.1.36.16) and alpha variant were the predominant circulating strains during the pre-delta (2^nd^ and 3^rd^) waves.

^†^ Days after COVID-19 diagnosis or onset of symptom

### Potential factors associated with developing pneumonia in pediatric patients with COVID-19

Sex, comorbidities, outbreak wave, fever, respiratory symptoms, gastrointestinal symptoms, S IgM levels, S IgG levels, hospitalization days, and intensive care unit admission were not significantly different between pediatric patients with (27.7%, 95% CI: 65.6%-78.3%) and without pneumonia (72.3%, 95% CI: 21.7%-34.4%) (S2 Table). No patients received a vaccine against COVID-19 and were not an immunocompromised host. Pediatric patients with pneumonia had a significantly lower rate of 5–18 years of age (53.6% vs. 71.2%; *p* = 0.017) and had significantly higher N IgG levels (5.9, IQR: 4.4–6.7 index vs. 4.3 index, IQR: 2.6–5.9 index; *p* = 0.0005) than those without pneumonia. All pediatric patients with COVID-19 and pneumonia had N IgG levels >1.5 index, which is considered high [16]. After adjusting for sex, comorbidities, immunocompromised status, respiratory symptoms, and gastrointestinal symptoms, N IgG levels were associated with pneumonia (OR: 1.29; 95% CI: 1.11–1.51; *p* = 0.001). Patients with pneumonia had N IgG tested at 5.9 (IQR: 4.4–6.7) median days after onset of COVID-19 symptoms.

## Discussion

Our study assessed the seroprevalence and seroconversion rates of COVID-19 in pediatric patients who visited a tertiary care center in Bangkok, Thailand, between September 2020 and February 2022 (Fig 1). The COVID-19 seroprevalence rate in pediatric patients increased during the Delta-Omicron period (70.6%) compared with the pre-Delta period (4.1%). This finding is consistent with previous hospital-based studies in children who had a higher COVID-19 seroprevalence rate in the wave that followed the first wave. These previous studies showed that pediatric patients who visited the hospital had COVID-19 seroprevalence rates of 0.27–2.9%, 8.4%, and 6.2–59.2% in the first, second, and fourth-fifth waves, respectively [17–20]. However, a study in India during the first and second waves reported a COVID-19 seroprevalence rate of 19.6% in hospitalized pediatric patients, which was markedly higher than in other studies, including ours [10]. The findings that almost half of the seropositive pediatric patients had multisystem inflammatory response syndrome may reflect this high seroprevalence rate [10]. The pooled COVID-19 seroprevalence rates in pediatric patients worldwide were 7.0%, 9.1%, 16.1%, and 25.6–63.9% in the first, second, third, and fourth-fifth waves, respectively, which were marginally higher than those observed in pediatric patients participating hospital-based studies [21–24]. In a subgroup of pediatric patients from Southeast Asia, COVID-19 seroprevalence was 57.2–81.8% during the fourth and fifth waves, which was higher than in other countries but in a similar range to our study and another study in Thailand [22, 25]. The fact that pediatric patients from the acute respiratory illness clinic were enrolled in our study, which focused primarily on patients under investigation or those with respiratory tract symptoms, may account for the marginally higher COVID-19 seroprevalence rate in pediatric patients during the Delta-Omicron period than that found in previous studies. In addition, our study confirmed that the Delta and Omicron variants had higher transmissibility than the original variants [26, 27]. Furthermore, our COVID-19 seroconversion rate was similar to that in a previous study [26].

In our study, independent risk factors for COVID-19 seropositivity included exposure to household members with confirmed COVID-19, being in the Delta-Omicron wave, respiratory symptoms, and being in an OPD (Table 1). In addition, exposure to household members with confirmed COVID-19 infection and the age group of 1–12 years old were an independent risk factor for COVID-19 seroconversion (Table 3). Living with a household member who has had COVID-19 and being in the Delta and Omicron waves were associated with COVID-19 seropositivity in previous studies [20, 21, 23, 28]. A household transmission study in Thailand during the pre-Omicron prominent wave demonstrated that factors associated with seropositivity in household contacts from a symptomatic index case were prolonged staying in the same room with the index case and participation in leisure activities with the index case [29]. In our study, information on demographic data, clinical data, and living activities of the index cases was not available. Previous studies demonstrated variable findings of age effect on seroconversion in pediatric patients with COVID-19 [25, 30–32]. These discrepancies might stem from different types of COVID-19 vaccination, SARS-CoV-2 viral load levels, SARS-CoV-2 strain, history of exposure to COVID-19 cases, and interval duration between antibody testing and COVID-19 diagnosis. In our study, the lower seroconversion rate in pediatric patients 1–12 years compared to pediatric patients <1 year might be from higher rates of exposure to COVID-19 cases or COVID-19 infection of the latter age during the Delta-Omicron predominant wave. A history of symptoms consistent with COVID-19 was associated with COVID-19 seropositivity in a previous study [28]. Our study reported a similar finding that respiratory symptoms, which were a frequent clinical presentation of patients with COVID-19, were associated with seropositivity. This study suggests that there is a higher rate of COVID-19 transmission in the community than in hospitals because pediatric patients in the OPD were independently at risk for COVID-19 seropositivity. The availability of screening tools for COVID-19, such as questionnaires and surveillance of COVID-19 testing, and hospital isolation policies may have contributed to the lower rate of COVID-19 in the hospital than in the community. Nonpharmaceutical preventive interventions have been shown to be an effective method of reducing COVID-19 transmission in hospitals in previous high-income country studies [33]. Systematic review studies demonstrated that school closures and in-school mitigations were associated with a decreased transmission rate of COVID-19 in the community [34]. We addressed this issue by including the national school opening period in the analysis. Our study demonstrated that serological blood tests during the national school opening period were not associated with COVID-19 seropositivity (data not shown). However, our study did not include data on types of school activities, the number of days each participant spent at school, and school preventive measures.

In this study, <6.5% of the pediatric patients with COVID-19 were seropositive for S IgM, S IgG, N IgG, and N total antibodies within 2 weeks of the onset of symptoms. In addition, we found that more than two thirds of pediatric patients with COVID-19 were seropositive for S IgM, S IgG, N IgG, and N total antibodies at least 2 weeks after the onset of symptoms. Previous studies in pediatric patients with COVID-19 reported a seropositivity rate ranging from 35% for S IgM, 25%-100% for S IgG, and 93% for N IgG during acute infection (<4 weeks) to 50% for S IgM, 63%-100% for S IgG, and 92%-100% N IgG during convalescent infection (4 weeks-6 months after disease onset) [5, 6, 35, 36]. Our study showed that the seropositivity rates of S IgM, S IgG, and N IgG were lower at < 2 weeks after the onset of symptoms, but they were comparable at >2 weeks, compared with other studies [35, 36]. However, our results are consistent with one study that reported low S IgM levels during acute and convalescent infections [5]. At 6 months postinfection, previous studies reported the seropositivity rates in pediatric patients with COVID-19 were 97% and 56–100% for S IgG and N IgG, respectively [35, 37]. Joshi *et al*. reported that SARS-CoV-2-specific IgG was still detectable for up to 500 days after diagnosis in pediatric patients with COVID-19 [5]. However, N IgG had a short half-life of 116 days as compared with S IgG in pediatric patients with COVID-19 [5, 6]. In our study, at 103, 191, 122, and 191 days after the onset of symptoms, S IgM, N IgG, S IgG, and N total antibodies, respectively, were still at detectable levels in pediatric patients with COVID-19. However, our study reported a more extended range for detected N IgG than S IgG in pediatric patients with COVID-19. The results of this study indicated that N IgG could be a good marker of having evidence of previous COVID-19 and independent factor associated with COVID-19 pneumonia. We anticipate that this would be beneficial in case of patients later develop symptoms suspecting MIS-C. Similar to our finding, previous studies in children with COVID-19 demonstrated that N IgG and S IgG might be useful in the diagnosis and disease stratification of MIS-C [10, 38]. In addition, it may be used as marker in study regarding long COVID-19 or other sequelae of COVID-19.

In this study, S IgG and N IgG seropositivity were associated with serological testing >14 days after the onset of symptoms in pediatric patients with COVID-19. These findings are consistent with a previous study, which showed that 100% of patients with COVID-19 had detectable S IgG and N IgG levels at intervals longer than 2 weeks [39]. In addition, our study showed that seropositivity of N total antibodies was associated with the OPD setting in pediatric patients with COVID-19. These results might reflect the higher rate of transmission in the community than in hospitals in the current study. Moreover, in our study, S IgM seropositivity was not likely to be detected during the Delta and Omicron waves in contrast to the pre-Delta wave. These results support a previous study, which showed that Omicron infection was less potent in inducing a humoral response than infection from the original strain [40]. Additionally, COVID-19 might be a secondary infection in patients with low S IgM levels, which less robustly elicits an IgM response as compared to a primary infection. The finding that S IgG seropositivity was also associated with the Delta-Omicron wave in the univariable analysis supports this hypothesis. In addition, we found that N IgG levels were associated with developing pneumonia in pediatric patients with COVID-19. This finding is consistent with previous studies in adults, which reported that high N IgG levels were associated with poor outcomes in patients with COVID-19 [16, 41]. Furthermore, approximately 30% of adult patients with COVID-19 and moderate-to-severe pneumonia had residual abnormal chest computerized tomography findings 1 year post COVID-19 that could lead to long-term pulmonary vascular sequalae [42]. Previous studies in pediatric patients with COVID-19 also reported an association between high S IgG and N IgG levels in hospitalized and symptomatic patients [6, 7, 43]. During the study period in Thailand, all patients with COVID-19 were admitted to the hospital or were in home isolation for at least 10–14 days after onset of symptoms or diagnosis according to Thai national guidelines for the management of COVID-19. Therefore, the association between N IgG levels and hospitalization could not be evaluated in pediatric patients with COVID-19 in our study.

This study has several limitations. First, not all patients without a history of COVID-19 underwent a nasal swab to determine SARS-CoV-2 by RT-PCR and had confirmation of serological testing with S IgM, S IgG, and N IgG. Second, not all patients with negative tests for N total antibodies (screening antibody test) were followed for seroconversion. Third, there were no verified sequencing data available for the SARS-CoV-2 variants from the different outbreak periods. Fourth, there were no clinical data on long-term follow-up, and thus long-term outcomes could not be assessed. Fifth, the study was conducted in a single tertiary care center. Therefore, the generalizability of our findings to other settings may not be applicable. Sixth, this study was conducted when the prevalence of other respiratory viruses was extremely low owing to the social distancing policy. Whether our results would be the same if the study is conducted when other respiratory viruses are co-circulating needs to be determined. Finally, the waning of the immunity observed in the Asian pediatric population study may also contribute to the underestimation of the seroprevalence in our study as we did not follow up with patients longitudinally [44].

## Conclusions

The COVID-19 seropositivity rate has continued to increase and persist in pediatric patients since the emergence of COVID-19. Pediatric patients who have been exposed to household members with COVID-19 and have respiratory symptoms during the COVID-19 outbreak should be suspected as having SARS-CoV-2 and tested for COVID-19. Regardless of whether a child has previously received mRNA- or protein-based COVID-19 vaccinations, high N IgG levels can be used as a biomarker for pediatric patients with COVID-19. Therefore, these patients with high N IgG level should be followed for acute and long-term complications. In addition, N IgG can be used as a screening tool in pediatric patients suspected of having a history of COVID-19 during an outbreak.

## Supporting information

S1 Fig Correlations of N IgG titer vs. S IgM titer, N IgG titer vs. S IgG titer, and S IgG titer vs. S IgM titer. (A) Correlation between nucleocapsid IgG titer and spike IgM titer. The vertical axis represents the nucleocapsid IgG titer. The horizontal axis represents spike IgM titer. (B) Correlation between nucleocapsid IgG titer and spike IgG titer. The vertical axis represents nucleocapsid IgG titer. The horizontal axis represents spike IgG titer. (C) Correlation between spike IgG titer and spike IgM titer. The vertical axis represents spike IgG titer. The horizontal axis represents spike IgM titer.
(TIF)

S1 Table Patients who had no history of COVID-19 with SARS-CoV-2 seropositivity. (DOCX)

S2 Table Potential factors associated with COVID-19 pneumonia. (DOCX)

S1 Data Data of all participants. (XLS)

S2 Data Data of participants who were followed for seroconversion. (XLS)
