# Supplementary figures and images for: Seroprevalence of SARS-CoV-2 infection in pediatric patients in a tertiary care hospital setting
Source: PLoS One. 2024 Sep 24;19(9):e0310860. doi: 10.1371/journal.pone.0310860 (PMC11421809; doi:10.1371/journal.pone.0310860)

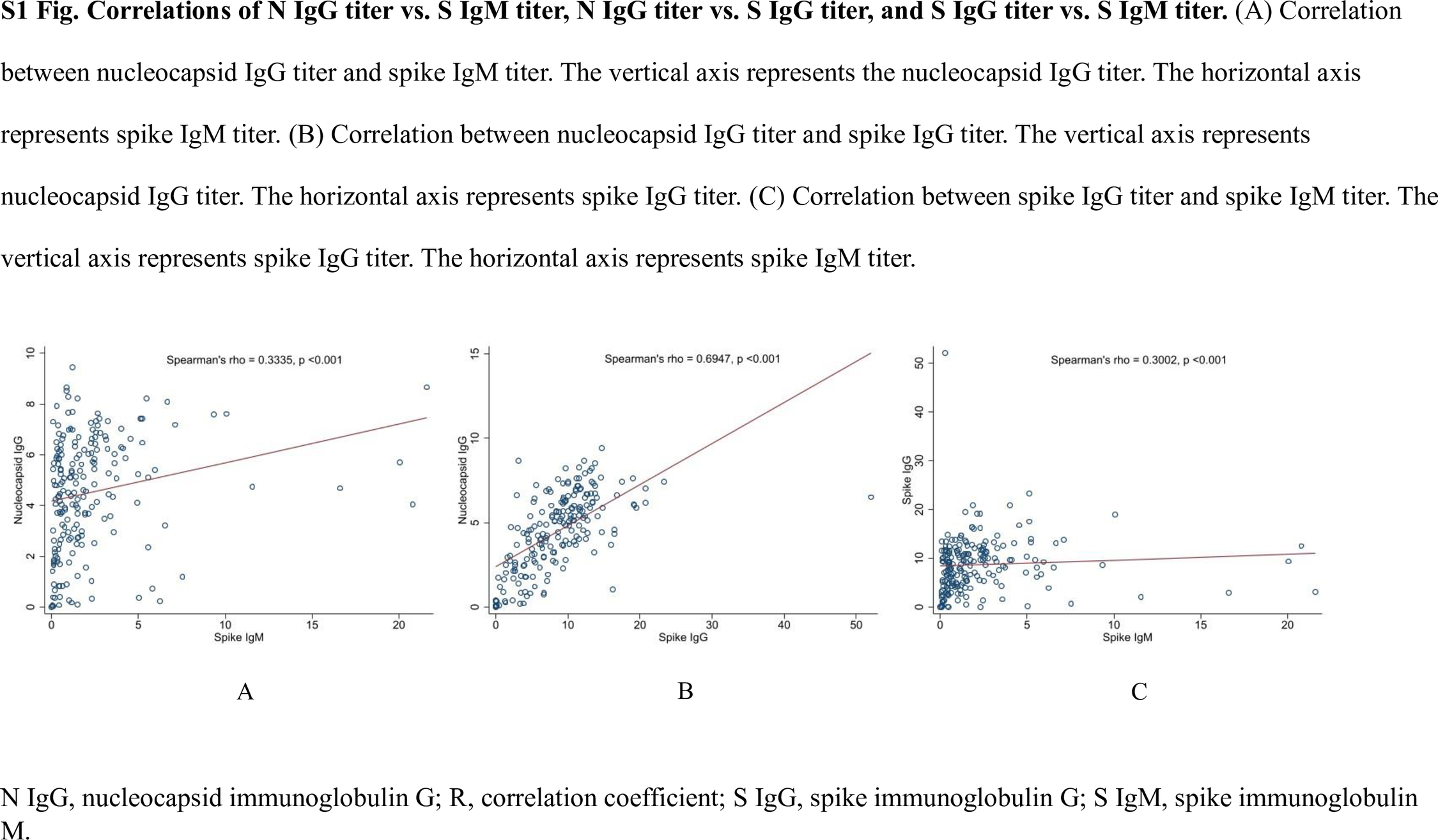

Supplement: S1 Fig — (A) Correlation between nucleocapsid IgG titer and spike IgM titer. The vertical axis represents the nucleocapsid IgG titer. The horizontal axis represents spike IgM titer. (B) Correlation between nucleocapsid IgG titer and spike IgG titer. The vertical axis represents nucleocapsid IgG titer. The horizontal axis represents spike IgG titer. (C) Correlation between spike IgG titer and spike IgM titer. The vertical axis represents spike IgG titer. The horizontal axis represents spike IgM titer. (TIF) [file pone.0310860.s001.tif]
