# Supplementary material for: Seroprevalence of SARS-CoV-2 infection in pediatric patients in a tertiary care hospital setting
Source: PLoS One. 2024 Sep 24;19(9):e0310860. doi: 10.1371/journal.pone.0310860 (PMC11421809; doi:10.1371/journal.pone.0310860)
Supplement: S1 Table — (DOCX) [file pone.0310860.s002.docx]

**S1 Table.** **Patients who had no history of COVID-19 with SARS-CoV-2 seropositivity.**

| N | Age (y) | Sex | IC | Vaccine dose | Days^†^ | PUI | Wave | Symptom | N total Ab | S IgM | S IgG | N IgG |
| --- | --- | --- | --- | --- | --- | --- | --- | --- | --- | --- | --- | --- |
| 1 | 3.3 | M | No | No | - | Yes | Pre-delta | Neg | Pos | - | Neg | - |
| 2 | 8.0 | M | No | No | - | Yes | Pre-delta | Neg | Pos | Neg | Pos | Pos |
| 3 | 12.8 | F | Yes | 2 | 6 | Yes | Pre-delta | Neg | Pos | Pos | Pos | Pos |
| 4 | 12.0 | M | No | 2* | 29 | Yes | Pre-delta | Neg | Pos | Pos | Pos | Pos |
| 5 | 14.5 | F | No | 2 | 10 | No | Pre-delta | Neg | Pos | Pos | Pos | Neg |
| 6 | 13.3 | F | No | 1 | 14 | No | Pre-delta | Yes | Pos | Pos | Pos | Neg |

F, female; IC, immunocompromised status; M, male; Neg, negative; N, number; N IgG, nucleocapsid IgG; N total Ab, nucleocapsid total antibody; Pos, positive; PUI, patient under investigation; SARS-CoV-2, severe acute respiratory syndrome coronavirus-2; S IgM, spike IgM; S IgG, spike IgG; y, year.

* Sinopharm COVID-19 vaccine.

^†^ Duration from the last dose of vaccine before enrollment.
