# Supplementary material for: Seroprevalence of SARS-CoV-2 infection in pediatric patients in a tertiary care hospital setting
Source: PLoS One. 2024 Sep 24;19(9):e0310860. doi: 10.1371/journal.pone.0310860 (PMC11421809; doi:10.1371/journal.pone.0310860)
Supplement: S2 Table — (DOCX) [file pone.0310860.s003.docx]

**S2 Table.** **Potential factors associated with COVID-19 pneumonia.**

|  | Total  (n = 202) | Pneumonia  (n = 56) | Non-pneumonia  (n = 146) | *P* | Univariable  OR (95% CI) | *P* | Multivariable  OR (95% CI) | *P* |
| --- | --- | --- | --- | --- | --- | --- | --- | --- |
| Age, n (%) |  |  |  | 0.017 |  |  |  |  |
| < 5 years | 68 (33.7) | 26 (46.4) | 42 (28.8) |  | Reference |  |  |  |
| 5-18 years | 134 (66.3) | 30 (53.6) | 104 (71.2) |  | 0.47 (0.25-0.88) | 0.019 |  |  |
| Male sex, n (%) | 105 (52.0) | 23 (41.1) | 82(56.2) | 0.055 | 0.54 (0.29-1.02) | 0.056 |  |  |
| Comorbidities, n (%) | 35 (17.3) | 14 (25.0) | 21 (14.4) | 0.074 | 1.98 (0.93-4.25) | 0.078 |  |  |
| Outbreak wave*, n (%) |  |  |  | 0.830 |  |  |  |  |
| Pre-delta | 49 (24.3) | 13 (23.2) | 36 (24.7) |  |  |  |  |  |
| Delta-omicron | 153 (75.7) | 43 (76.8) | 110 (75.3) |  |  |  |  |  |
| Fever, n (%) | 101 (50.0) | 25 (44.6) | 76 (52.1) | 0.346 |  |  |  |  |
| Respiratory symptom, n (%) | 165 (81.7) | 50 (89.3) | 115 (78.8) | 0.084 | 2.25 (0.88-5.72) | 0.090 |  |  |
| GI symptom, n (%) | 20 (9.9) | 9 (16.1) | 11 (7.5) | 0.069 | 2.35 (0.92-6.02) | 0.075 |  |  |
| S IgM, median (IQR) | 1.26 (0.48-2.53) | 1.41 (0.47-3.14) | 1.20 (0.51-2.46) | 0.350 |  |  |  |  |
| S IgG, median (IQR) | 8.72 (4.8-11.6) | 9.85 (6.11-13.1) | 8.04 (4.27-11.50) | 0.021 |  |  |  |  |
| N IgG, median (IQR) | 4.81 (2.81-6.24) | 5.85 (4.42-6.75) | 4.29 (2.64-5.87) | 0.0005 | 1.29 (1.11-1.51) | 0.001 | 1.29 (1.11-1.51) | 0.001 |
| Hospital stays, n (%) | 10 (9.0-12.0) | 10.5 (9.0-13.0) | 10 (9.0-12.0) | 0.485 |  |  |  |  |
| ICU admission, n (%) | 1 (0.5) | 1 (1.9) | 0 (0.0) | 0.101 |  |  |  |  |

95% CI, 95% confidence interval; GI, gastrointestinal; IPD, inpatient department; IQR, interquartile range; n, number; N IgG, nucleocapsid immunoglobulin G; S IgG, spike immunoglobulin G; S IgM, spike immunoglobulin M; OR, odds ratio; y, year;

^†^ COVID-19 vaccine (Pfizer or Sinopharm).

* Original strain (B.1.36.16) and alpha variant were the predominant circulating strains during the pre-delta (2^nd^ and 3^rd^) waves.
